# Unravelling Immunoglobulin G Fc *N*-Glycosylation: A Dynamic Marker Potentiating Predictive, Preventive and Personalised Medicine

**DOI:** 10.3390/ijms19020390

**Published:** 2018-01-29

**Authors:** Alyce Russell, Eric Adua, Ivo Ugrina, Simon Laws, Wei Wang

**Affiliations:** 1 School of Medical and Health Sciences, Edith Cowan University, Joondalup 6027, Australia; a.russell@ecu.edu.au (A.R.); eadua@our.ecu.edu.au (E.A.); s.laws@ecu.edu.au (S.L.); 2 Department of Twin Research & Genetic Epidemiology, King’s College London, London SE1 7EH, UK; iugrina@genos.hr; 3 Faculty of Pharmacy and Biochemistry, University of Zagreb, 10000 Zagreb, Croatia; 4 School of Biomedical Sciences, Faculty of Health Sciences, Curtin Health Innovation Research Institute, Curtin University, Bentley 6102, Australia; 5 Co-Operative Research Centre for Mental Health, Carlton South 3053, Australia; 6 Key Municipal Laboratory of Clinical Epidemiology, Capital Medical University, Beijing 100054, China; 7 Taishan Medical University, Taian Shi 271016, China

**Keywords:** immunoglobulin G, glycosylation, effector function, biomarker, environmental factors, glycomics

## Abstract

Multiple factors influence immunoglobulin G glycosylation, which in turn affect the glycoproteins’ function on eliciting an anti-inflammatory or pro-inflammatory response. It is prudent to underscore these processes when considering the use of immunoglobulin G *N*-glycan moieties as an indication of disease presence, progress, or response to therapeutics. It has been demonstrated that the altered expression of genes that encode enzymes involved in the biosynthesis of immunoglobulin G *N*-glycans, receptors, or complement factors may significantly modify immunoglobulin G effector response, which is important for regulating the immune system. The immunoglobulin G *N*-glycome is highly heterogenous; however, it is considered an interphenotype of disease (a link between genetic predisposition and environmental exposure) and so has the potential to be used as a dynamic biomarker from the perspective of predictive, preventive, and personalised medicine. Undoubtedly, a deeper understanding of how the multiple factors interact with each other to alter immunoglobulin G glycosylation is crucial. Herein we review the current literature on immunoglobulin G glycoprotein structure, immunoglobulin G Fc glycosylation, associated receptors, and complement factors, the downstream effector functions, and the factors associated with the heterogeneity of immunoglobulin G glycosylation.

## 1. Introduction

**Figure 1:**
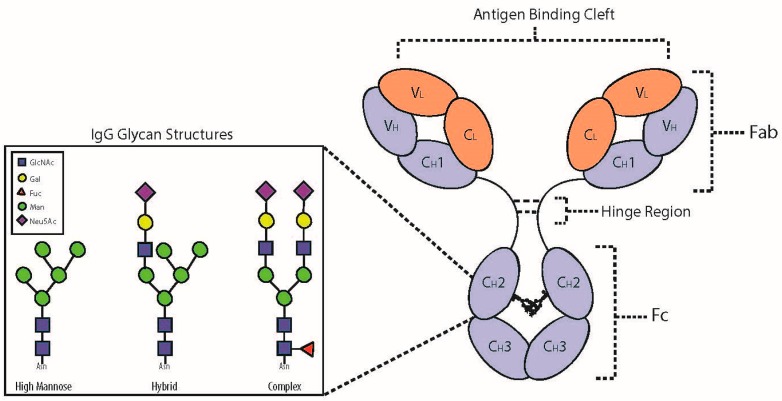
IgG and the associated *N*-glycan structures. Consisting of both heavy chains (‘_H_’) and light chains (‘_L_’), the IgG glycoprotein has two domains that infer different properties; the fragment antigen-binding (Fab) and fragment crystallisable (Fc) domains. The Fab and Fc are connected by a hinge region containing disulphide bonds, which differ depending on the IgG subclass. The Fab domain is responsible for recognising and binding antigen. The Fc domain contains two glycans attached to conserved regions of the C_H_2. The Fc domain elicits effector functions by binding Fc receptors (FcRs) on natural killer and other inflammatory cells. Changes to the attached glycan moieties, simplified as the three glycan structural types in the side figure, can significantly alter effector functions of the IgG glycoprotein. GlcNAc—*N*-acetylglucosamine. Gal—galactose, Fuc—core fucose, Man—mannose, Neu5Ac—*N*-acetylneuraminic acid (sialic acid).

Immunoglobulin G (IgG) is an important effector glycoprotein linking the innate and adaptive branches of the immune system. It has the ability to exert both anti-inflammatory and pro-inflammatory responses throughout the body, which are triggered by antigen recognition, and are dependent on its affinity for a number of different activating or inhibitory fragment crystallisable receptors (FcRs) and complement factors. This process mediates key effector functions, including pathogen clearance, antibody-dependent cell cytotoxicity (ADCC), and complement-initiated inflammation, all with both beneficial and detrimental effects depending on the premise of the IgG activity. For example, during primary bacterial infection, IgG can initiate opsonisation through complement activation and phagocytosis of the bacterial cells by macrophages, monocytes and neutrophils, as well as neutralise endotoxins and exotoxins [1,2,3]. These beneficial effects are well-established and have been harnessed in the form of intravenous immunoglobulin therapy in immunodeficient individuals [4]. On the contrary, there are examples where these effects are detrimental, such as in rheumatoid arthritis patients [5,6,7]. In this instance, IgG is thought to tandemly bind synovial cells and mannose-binding lectin (MBL), resulting in the initiation of the lectin complement cascade and secondary damage of surrounding tissues within the synovial joints [5,6,7]. These immune responses are largely modulated by the fragment crystallisable (Fc) domain of the IgG glycoprotein (Figure 1).

It has been established that the IgG Fc sugar moieties, hereon known as *N*-glycans, affect the affinity of the Fc domain for a number of different FcRs, ultimately initiating different cellular events that lead to an array of inflammatory responses [8]. The glycosylation of the IgG Fc domain may be a predesigned outcome of the producing B cell, and variation in IgG glycosylation has physiological significance [8,9]. Different inflammatory factors influence B cells during activation and differentiation, and can modulate the glycosylation of secreted IgG without disturbing the general cellular glycosylation machinery [10]. Furthermore, glycosylation changes in the IgG glycome are thought to be fairly stable over short periods of time, and modifications can result from biological and chronological age [11], as well as altered cellular environment and disease status [9,12].

Therefore, the *N*-glycan moieties of IgG represent intermediate phenotypes of subclinical and chronic disease. They are a link between the genetic makeup of our cells and the cellular environment, which is largely under the influence of our habits and daily routines [12]. With so much interest in utilising the IgG glycome in the pursuit for predictive, preventive and personalised medicine, this begs the question: are the genetic changes more significant when it comes to elucidating aberrant IgG glycosylation or are they irrelevant when compared with the contribution of the cellular environment? This paper reviews the current literature on the IgG glycoprotein and its FcRs, as well as the factors associated with altered IgG glycosylation.

## 2. IgG Glycoprotein Structure and Function

The immunoglobulins are a group of plasma glycoproteins, among which IgG is the most abundant [6]. The protein portion of IgG consists of four polypeptide chains, two identical light chains and two identical heavy chains (Figure 1), which are held together by inter-peptide disulphide bonds [13]. There are two types of light chains, kappa and lambda, with their genes located in 2q12 and 22q11, respectively, and one type of heavy chain with its gene located in 14q32 [14]. Intra-peptide disulphide bonds also exist and cause the formation of loops, which link the anti-parallel β-sheets in the tertiary structure of IgG [13]. There are two functionally distinct domains of the IgG glycoprotein: the fragment antigen-binding (Fab) and the Fc domains. The Fab domain contains the variable (V_L_ and V_H_) and constant (C_L_ and C_H_1) regions of the light and heavy chains. These form the antigen-binding sites, with the variable regions recognising and distinguishing specific molecular structures of antigens [13]. On the contrary, the Fc domain contains constant regions (C_H_2 and C_H_3) and mediates key effector functions [15].

Antigens are diverse; thus, the repertoire of IgG Fab domains must also be diverse to target these ubiquitous molecular structures. This is achieved during B cell development through V(D)J shuffling (also known as somatic recombination) of the multiple gene segments scattered along the locus of a given IgG polypeptide [13]. Introduced somatic mutations are also important for Fab polyclonality [16]. The variable regions V_H_ and V_L_, primarily responsible for antigen specificity, are made up of highly variable subregions involved in the antigen-binding activity [16]. These are known as complementary-determining regions (CDR1 to CDR3), and are flanked by less variable regions known as framework regions (FR1 to FR4). Proximal to the hinge on the Fab domain are the constant regions C_L_ and C_H_1. The antigen-binding sites are the culmination of these subregions. Nevertheless, even with the proper recognition of specific antigens, the IgG cannot mount an effector response without the glycosylation of the Fc domain [17].

There are four different subclasses of IgG (IgG1 to IgG4). These subclasses arise from alternative amino acid sequences translated from the singular heavy chain gene (14q32) [13] and they differ in the length of their hinge region and the number of disulphide bonds present. These variations result in an altered affinity for a number of different FcRs or complement factors and, therefore, different effector functions [16]. Moreover, the effector functions are influenced by the *N*-glycan moieties attached to both C_H_2 regions. These interact with each other to change the conformation of the Fc domain (Figure 1). It is possible that a single altered monosaccharide on an *N*-glycan moiety can lead to a conformational change that is inhibiting rather than activating, and may even change the type of FcR or complement factor bound by the Fc domain.

Though beyond the scope of this review, it should be noted that more recently the importance of Fab glycosylation, which is evident on 15–20% of circulating IgG, has also been reported [16,18]. Moreover, three recognition sites specific for *O*-glycosylation exist on the hinge region of IgG3. The IgG3 subclass constitutes approximately 8% of the IgG glycoprotein pool, with only 10–13% expected occupancy of any of these three *O*-glycosylation sites [19].

## 3. *N*-Glycosylation of the Fc Domain

An *N*-glycan is covalently attached to the side chain nitrogen atom of the highly conserved asparagine (Asn)-297 of both heavy chains (Figure 1). This occurs at the recognition sequence Asn–X–Serine (Ser)/Threonine (Thr), located in the C_H_2 domains, whereby X is any amino acid except proline [6,12]. The majority of these *N*-glycans are complex-type biantennary structures (Figure 1); however, a high degree of heterogeneity exists due to the presence of different monosaccharides [12]. In total, over 36 IgG glycoforms have been identified [9,20,21], and the two *N*-glycan moieties that attach to a single IgG in the Fc domain may vary, leading to greater variability in effector function [22]. These *N*-glycans are thought to not only stabilise the quaternary structure of the IgG Fc, but also contribute to recognition of the target FcRs [6,23]. Importantly, IgG lacking Fc *N*-glycans may have a conformation which may hinder FcR binding [12,17]. This is due to the Fc glycan moieties interacting with and stabilising the C’E loop, the region of the C_H_2 domains containing the recognition sequence [1]. Though on the contrary, recent research has demonstrated the capacity for therapeutic aglycosylated IgG to be engineered with Fc binding affinity [24].

**Figure 2:**
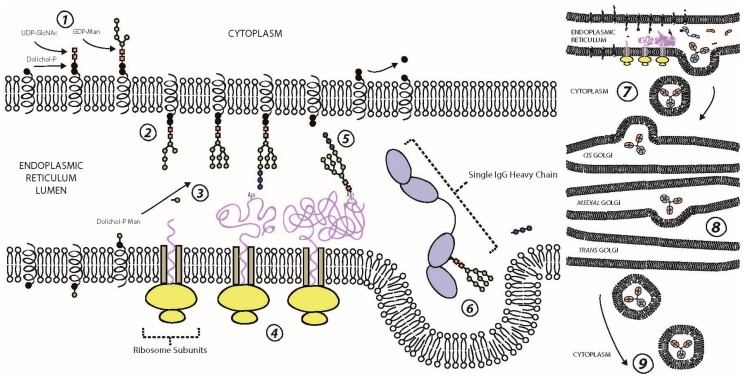
Biosynthesis of the IgG glycoprotein. ① *N*-glycan biosynthesis begins on the endoplasmic reticulum membrane where two *N*-acetylglucosamines (UDP-GlcNAc) and five mannose (GDP-Man) sugar nucleotides contribute the given monosacchairdes to a dolichol-phosphate (Dolichol-P) donor molecule on the cytoplasmic side. ② The whole Dolichol-P attached *N*-glycan is flipped so it is on the luminal side of the endoplasmic reticulum. ③ Dolichol-P flips individual sugar nucleotides from the cytoplasm to the lumen. ④ In tandem, the ribosomes biosynthesise the polypeptide structure of the IgG. ⑤ Oligosaccharyltransferase transfers the *N*-glycan moiety from Dolichol-P to asparagine (Asn) 297 on the growing polypeptide. ⑥ The three terminal *N*-acetylglucosamine sugar nucleotides are removed over two steps, following folding and assembly of the nascent IgG, and this signals the transfer of the IgG from the endoplasmic reticulum to the Golgi apparatus. ⑦,⑧ The IgG components move through the Golgi apparatus, where different glycosyltransferases and glycosylhydrolases add and remove respectively, different sugar nucleotides to the glycan moiety. ⑨ Following excretion from the *trans* Golgi, the final IgG glycoprotein either secreted from the B-cell lymphocyte or attached to the plasma membrane of the B-cell to become a B-cell receptor (BCR).

*N*-glycans are biosynthesised sequentially (Figure 2) and in tandem to protein biosynthesis, commencing on the membrane of the endoplasmic reticulum (ER). Here, sugar nucleotides assemble onto a dolichol pyrophosphate donor molecule on the cytoplasmic side of the ER membrane to form a mannose (Man)_5_ *N*-acetylglucosamine (GlcNAc)_2_ oligosaccharide [16]. The structure is subsequently inverted to be on the luminal side of the membrane and extended by a further four Man and three glucose (Glc) sugar nucleotides before the dolichol-bound Glc_3_Man_9_GlcNAc_2_ oligosaccharide, termed a precursor *N*-glycan, is transferred to an Asn recognition sequence by oligosaccharyltransferase [12]. The HC and LC polypeptides are then folded and the nascent IgG assembled into its respective form. Following removal of the three terminating Glc via two trimming steps, the glycoprotein with attached Man_9_GlcNAc_2_ oligosaccharides is transferred to the *cis* portion of the Golgi apparatus for further glycoprocessing [25,26]. Different glycosylhydrolases and glycosyltransferases remodel the branched IgG glycan structure throughout the glycoprocessing steps, from the *cis* Golgi to the *trans* Golgi [27]. Variation in these enzymatic processes results in diversification of the IgG *N*-glycome composition.

The quaternary structure of the IgG Fc is thought to limit accessibility of the *N*-glycan moieties to glycosyltransferases in the Golgi, thus limiting the variability of galactosylation and sialylation [6]. It has also been argued that intramolecular interactions, which increase as the *N*-glycan moieties are extended, restrict glycoprocessing [28]. This constraint to diversify may be essential for the regulation of effector functions. Subsequently, the final glycosylated IgG is secreted into the blood serum or assimilated into the plasma membrane to form B cell receptors (BCRs).

**Figure 3:**
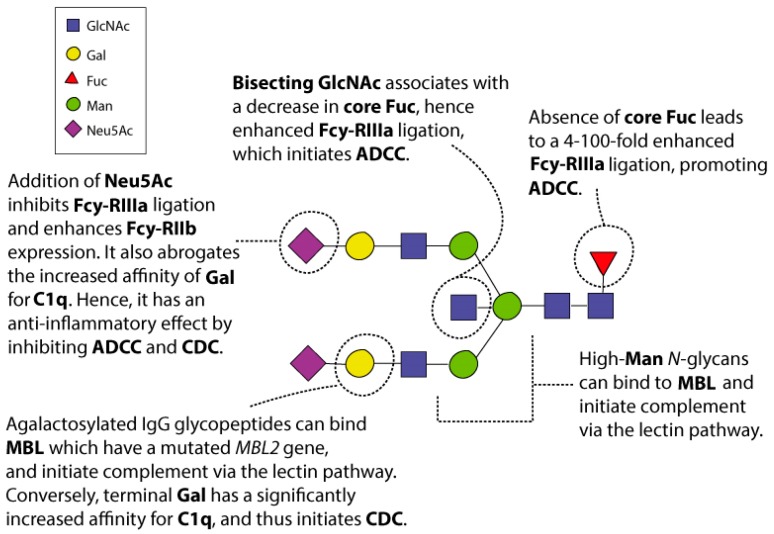
A picture summary of altered IgG glycosylation and its downstream effects. GlcNAc—*N*-acetylglucosamine. Gal—galactose, Fuc—core fucose, Man—mannose, Neu5Ac—*N*-acetylneuraminic acid (sialic acid), ADCC—antibody-dependent cell cytotoxicity, CDC—complement-dependent cytotoxicity.

The resulting *N*-glycans on a given IgG Fc are a predesigned outcome that depends on the relative expression of the genes that encode the specialised enzymes, particularly the glycosyltransferases, and the availability of the sugar nucleotides within the producing B cell lymphocyte [13]. These are clearly affected by age, pregnancy, hormones, cytokines, bacterial DNA, and food metabolites [13]. Differences in glycosylation pattern result in altered binding affinities for the many FcRs and complement factors, which influence effector response [6,13,17]. A shift towards certain types of IgG *N*-glycans has been reported for a number of diseases and conditions, including rheumatoid arthritis [29,30], metabolic syndrome and type 2 diabetes mellitus [9,31], inflammatory bowel disease [9], systemic lupus erythematosus [32], hypertension [33], various cancers [9,34], and more recently neurological disorders such as Alzheimer’s disease and progressive mild cognitive impairment [35], multiple sclerosis [36], and Parkinson’s disease [37]. This makes the study of the *N*-glycan moieties biologically important in terms of the physiological activity of IgG in the human body. Indeed, even alterations to the IgG *N*-glycan moieties that appear structurally minute can significantly change its affinity to a number of different FcRs and complement factors (summarised in Figure 3).

The addition of *N*-acetylneuraminic acid (Neu5Ac), more commonly referred to as sialic acid, to the terminating end of the IgG Fc *N*-glycan has a similar anti-inflammatory effect to the addition of core fucose (Fuc), i.e., fucosylation of the Asn-attached core GlcNAc [23,38]. Sialylated IgG Fc *N*-glycans may have a lower affinity for FcγRIIIa on natural killer cells leading to an anti-inflammatory effect [6]. Sialylated, particularly α2-6 sialylated, IgG Fc *N*-glycans exert their anti-inflammatory effect by binding to a type II FcR known as dendritic cell-specific intercellular adhesion molecule-3-grabbing non-integrin (DC-SIGN), a C-type lectin which is present on dendritic cells [15]. Sialylation results in increased conformational flexibility of IgG Fc with a preference to engage these types of FcR [15], though the target FcRs of sialylated IgG have more recently been debated [23]. For example, an FcR-independent reduction in pro-inflammation also occurs due to inhibition binding of IgG Fc to C1q [16,39]. Interestingly, it has been found that B-cell-independent sialylation of IgG exists, as it can be regulated by the release of ST6Gal1 from the cells lining central veins in the liver, and by circulating nucleotide sugar donor CMP-Neu5Ac, which degranulating platelets partially contribute to [40]. Autoantibodies produced during autoimmune or inflammatory diseases frequently lack terminating galactose (Gal), and therefore Neu5Ac, which decreases the inhibition of ADCC [15]. In vitro and in vivo studies have also demonstrated that autoantibodies lacking sialylation enhance osteoclastogenesis, providing a possible mechanism for the drastically increased levels of agalactosylated IgG structures found in rheumatoid arthritis patients [41]. However, it has not been reported whether its absence leads to such a high increase in ADCC, as seen with the lack of core Fuc.

Out of all the *N*-glycosylation traits, core Fuc may be the most important in terms of effect modification in affinity to FcRs [27]. A lack of core Fuc leads to a 4 to 100-fold increase in ADCC due to increased binding affinity for FcγRIIIa and FcγRIIIb, making these IgG more pro-inflammatory [23,27,42,43]. Conversely, the presence of Fuc on the Asn-attached core GlcNAc of the IgG Fc *N*-glycan has an anti-inflammatory effect by inhibiting the binding of type I FcRs, particularly FcγRIIIa [15,42]. FcγRIIIa are primarily expressed on the surface of natural killer cells [8] and have a role in initiating ADCC [42]. Previous studies [8,9] have shown that the vast majority of IgG Fc are comprised of at least one core fucosylated *N*-glycan moiety, hence they are designed to be less efficient at ADCC activation subsequent to many IgG binding to an antigen (via the antigen-binding sites; see Figure 1). However, structural analysis of the IgG Fc/FcγRIIIa complex has demonstrated that specific *N*-glycans on FcγRIIIa at Asn-162 may also influence the effect of the absence of core Fuc [23,44], due to direct glycan-glycan interactions [6]. The presence of a bisecting GlcNAc has also been associated with enhanced ADCC activation via FcγRIIIa binding [45] but to a lesser degree than the absence of core Fuc [42].

It was first suggested that Man-rich *N*-glycans have an increased affinity for MBL, which initiates the lectin complement cascade via opsonisation of the target cells by IgG [5]. Both Man and GlcNAc (GlcNAc to a lesser degree than Man) are suggested to be prominent ligands for MBL, and are usually found on the surface of pathogens, such as bacteria and viruses [46]. Other monosaccharides, such as Neu5Ac and Gal, normally decorate mammalian glycoproteins and have undetectable affinity for MBL [5,46]. This enables the specific recognition of *N*-glycans on pathogen cell surfaces. However, this innate immunity system can malfunction, and has been implicated in many disease processes due to an increase in agalactosylated (Man-rich or GlcNAc terminating) *N*-glycans. Importantly, an increase in agalactosylated IgG is associated with advancing age, thus we may be more at risk for developing immune-related diseases as we age [8,47,48]. Therefore, agalactosylated IgG are considered pro-inflammatory.

In vitro studies have demonstrated a link between agalactosylated IgG and increased MBL binding [5,22]. After multiple recent in vivo studies, however, it is still unclear whether the mechanism for complement activation is via MBL, as first hypothesised or another complement factor [6]. For example, MBL-null and wild-type mice with agalactosylated IgG were found to have no difference in potential to activate inflammation [22]. Indeed, evidence suggests that galactosylation in the absence of sialylation significantly increases complement-dependent cytotoxicity via increased C1q binding, activating the classical complement cascade [39,49], highlighting another IgG glycan-modulated complement pathway. Of note, *MBL2* polymorphism may also be required for the association of agalactosylated IgG and activation of the lectin complement cascade [29].

## 4. IgG Fc Structure and Function

The IgG Fc domain has the ability to bind to a range of different FcRs and complement factors, resulting in distinct effector and immunomodulatory pathways. Monomeric IgG mediates these effects, particularly by binding to FcγRI [16,50]. Nevertheless, the formation of immune complexes (IC) is a prerequisite for efficient FcR binding [50]. FcR binding requires the presence of the Fc *N*-glycan moieties; however, in some cases, the *N*-glycans themselves may not interact with the FcRs as in the case of type II FcRs for example [15]. This suggests that *N*-glycan moieties modulate the FcR affinity by altering the conformational state of the Fc domain.

There are two dominant conformational states that the Fc domain may exhibit, and two structurally distinct sets of FcRs with selective abilities to engage the Fc domain. Type I FcRs belong to the immunoglobulin receptor superfamily, and include: the activating FcRs, FcγRI, FcγRIIa, FcγRIIc, FcγRIIIa, and FcγRIIIb; and the inhibitory FcR, FcγRIIb [15,16,51]. Specifically, these FcRs bind to the Fc domain of IgG in the open conformation, neighbouring the hinge region with a stoichiometry of 1:1 [1,15,52]. Whereas type II FcRs belong to the C-lectin receptor family, and include: CD209, which is also known as DC-SIGN; and CD23, which is an IgE FcR (FcεRII) that also has weak binding affinity for IgG [15]. These FcRs bind to the Fc domain in the closed conformation, which occludes the type I FcR binding site and permits type II FcR attachment in the region where C_H_2 and C_H_3 join with a stoichiometry of 2:1 [15]. The cellular events following binding of these FcRs are important, and the different effector functions elicited, such as the transcription of cytokines, may have a direct impact on the future glycosylation of IgG (summarised in Table 1). It should be noted that IgG Fc also ligates neonatal FcR (FcRn), which increases the half-life of serum IgG [4,22].

### Coengagement of Activating and Inhibitory FcRs

The activating and inhibitory FcRs exist on many different cell types in varying quantities, depending on the primary physiological response of the cell. IC that are composed of differing Fc conformations will simultaneously interact with either activating or inhibitory type I or type II FcRs on a single immune cell [15]. Activating FcRs are present on all myeloid cells, with cross-linking resulting in sustained effector response [38]. Catalysed by tyrosine kinases, activating FcRs mediate IgG immunomodulatory response by undergoing phosphorylation of an immunoreceptor tyrosine-based activation motif (ITAM) present in its cytoplasmic tail [16]. Phosphorylated ITAM becomes a binding site for Src homology 2 (SH2) [61], and this complexation triggers a signalling cascade that begins with the activation of Syc kinases. Activated Syc kinases phosphorylate and activate phosphatidylinositol 3-kinase (PI-3K). This then stimulates other signalling intermediates such as anti-apoptotic kinase (Akt), mitogen activated protein kinase (MAPK), and phospholipase C-gamma (PLC-γ). In particular, activated PLC-γ influences the dissociation of phosphatidylinositol 4, 5-bisphosphate (PIP2) into inositol 1, 4, 5-trisphosphate (IP3) and diacylglycerol (DAG). These two metabolites perform distinct roles in the signalling pathway. While DAG activates protein kinase C (PKC), IP3 triggers Ca^2+^ mobilisation from tissues and promotes cell proliferation [61].

Conversely, inhibitory FcRs also exist and act to dominantly dampen activating the immunomodulatory response [38]. Inhibitory FcRs, particularly FcγRIIb, negatively mediate this response by utilising a phosphorylated immunoreceptor tyrosine-based inhibitory motif (ITIM) on its cytoplasmic tail, triggering a downstream signalling pathway [16]. This signalling is fuelled by SH2 phosphatases, such as SH2 inositol 5-phosphate (SHIP) 1 & 2, SHP-1, and phosphatase and tensin homologue (PTEN), that act to dephosphorylate certain specific proteins in the signalling cascade. For example, SHIP is involved in the hydrolysis of phosphatidylinositol-3, 4, 5-triphosphate (PIP3) into PIP2. Importantly, SHIP negatively regulates PKC and MAPKs, thereby inhibiting cell proliferation [62].

Although the inhibitory pathway is thought to be dominant, it is the ratio between the activating and inhibitory signalling pathways on a given cell that determines the immunomodulatory outcome [4,15,16,38]. Thus, the FcR are ‘designed’ in such a way to prevent inflammation. Coengagement of activating and inhibitory FcRs leads to tyrosine phosphorylation via LYN kinase, recruitment of SH2-domain containing SHIP, and inhibition of ITAM-triggered Ca^2+^ mobilisation and cellular proliferation [51].

## 5. Fc Effector Functions

As stated earlier, the Fc domain of IgG is responsible for the glycoproteins’ key effector functions. These are initially triggered following antigen recognition and the binding of one of the many FcRs or complement factors [15], and can be grouped into being either pro-inflammatory or anti-inflammatory in nature. The process mediates pathogen neutralisation, opsonization, and clearance, as well as ADCC and complement-initiated inflammation, all with both beneficial and detrimental effects depending on the premise of the IgG activity. Moreover, more than one of these effector functions can occur in a given cell at any particular time, due to up-stream factors that promote simultaneous outcomes. Summarised in Table 2 are those effector functions pertaining to malfunctions in IgG, particularly with regard to autoimmune and inflammatory-related diseases.

## 6. The Complement Cascade

The complement cascade is a major component of the innate immune system and it is believed it evolved before the development of the adaptive immune system [50]. There are three initiation pathways for the complement cascade: the classical pathway, the alternative pathway, and the lectin pathway. The key component of the complement cascade is the activation of C3, either directly or indirectly. C3 harbours a reactive thioester moiety that facilitates the attachment of C3 to molecules and cell surfaces, thus allowing them to become targets for other complement receptors [50,63]. Specifically, it is the cleavage component of C3, anaphylatoxin C3b, which initiates the complement cascade. Following this cleavage, a number of events occur, including the conversion of C3-convertase to C5-convertase [6,63]. The cleavage of C5, a C3 homolog that lacks the reactive thioester, initiates inflammation through the formation of membrane-attack complexes (MACs) that target the cell surface [63]. These MACs contain the C5b component, along with C6, C7, C8, and many copies of C9, which are recruited sequentially, and their role is to perforate the cell membrane and cause lysis [63].

Glycosylation of the IgG Fc domain is associated with activation of all three complement cascades. Namely, the addition of terminal Gal is associated with significantly increased affinity for the C1q component of the classical pathway [49]. The addition of terminal Neu5Ac, however, abrogates the effect of increased affinity of galactosylated IgG Fc for the C1q component, leading to an FcR-independent anti-inflammatory response [39]. On the contrary, a decrease in terminal Gal or increase in high-Man glycoforms may be associated with an increased affinity for MBL of the lectin pathway [50] and C3b of the alternative pathway [16].

## 7. Loci Associated with Aberrant IgG Glycosylation

A number of loci have been identified as being associated with aberrant glycosylation. Importantly, these have shown a clear directional effect with either increases or decreases in the relative abundance of particular *N*-glycan moieties. The first locus found to be associated with aberrant plasma glycosylation was hepatocyte nuclear factor 1α (*HNF1α*), a master regulator of the expression of the fucosyltransferase genes, fucosyltransferase-6 (*FUT6*) and *FUT8*, that influence multiple stages in fucosylation [9]. Particularly for IgG, *FUT8* has been found to be associated with core fucosylation of the *N*-glycan moieties in Caucasian populations, and therefore, is important for the regulation of the immune response [9].

In addition, many other loci were identified as being associated with IgG glycosylation. Of those specifically linked to glycosylation, sialyltransferase 6 (*ST6GAL1*), β-1,4-galactosyltransferase (*B4GALT1*), and mannosyl (β-1,4)-glycoprotein β-1,4-*N*-acetylglucosaminyltransferase (*MGAT3*) encode genes for glycosyltransferases [9,66]. Whereas genes previously associated with other diseases, such as autoimmune diseases and haematological cancers, and later identified as being associated with IgG glycosylation, include *IL6ST-ANKRD55*, *IKZF1*, *ABCF2-SMARCD3*, *SUV420H1*, *SMARCB1-DERL3* and *SYNGR1-TAB1-MGAT3-CACNA1I* [9,66]. Though this study contained participants from four populations (Orkney Islands in the UK, Vis and Korcula Islands in Croatia, Northern Sweden, and The Netherlands), one for validation (The Netherlands), there is still a considerable gap in knowledge regarding the association of specific loci and IgG glycosylation, and to what extent this can impact the final *N*-glycan moiety when compared with environmental factors.

## 8. Environmental Factors Associated with Aberrant IgG Glycosylation

Further to the abovementioned genetic alterations, the cellular environment is associated with aberrant glycosylation, which strongly influences inflammatory properties. The IgG glycome is malleable as it is reliant on the expression levels of enzymes such as glycosyltransferases and glycosylhydrolases, and the abundance of sugar nucleotide donors, which in turn are epigenetically regulated within the producing B/plasma cells. Further, the IgG *N*-glycome is considered a link between the genetic makeup of cells and the cellular environment. Therefore, in theory, one can change their IgG *N*-glycan composition through modification of lifestyle choices, such as participating in certain activities (i.e., reduced/no smoking and alcohol, and increased exercise) and eating healthy.

Aside from disease presence, altered plasma protein glycosylation has been linked to gender, age, smoking status, body mass index (BMI), plasma lipid profile parameters (high-density lipoprotein (HDL), low-density lipoprotein (LDL), total cholesterol (TC) and triglyceride (TG) levels), blood pressure, fasting blood glucose (FBG), certain medications and diet [67,68]. Several factors have been further explored in association with IgG glycosylation, which could drastically affect the affinity of IgG Fc for the aforementioned FcRs and complement factors.

As stated earlier, one of the most profound factors associated with IgG glycosylation, particularly increasing agalactosylation, is ageing. The IgG glycome explains between 23.3–58% of the variance in age [11,48,69]. Numerous “GlycanAge” concept studies have been able to explain age in different populations [11,47,48,70] using either blood stains or plasma [11,69,71]. They have the potential to not only inform individuals of their “biological age”, but also give incentive to improve overall health. Although the concept of ageing can be the culmination of unfavourable levels of multiple factors, the translation of glycomics (i.e., the system-wide study of the relative abundance of glycan moieties) for use in predictive, preventive and personalised medicine is becoming a reality [12].

Gender [11,48] and hormone levels [72,73] are also associated with notable changes to the IgG Fc glycome. Particularly, these factors affect IgG Fc galactosylation and sialylation, with evidence of cyclical changes, such as in the menstrual cycle [72]. Although not the focus of this review, it should be noted that IgG Fab glycosylation is also associated with changing hormones in pregnancy [74]. It has been suggested that oestrogens may be responsible for modulating IgG Fc galactosylation in both women and men, with the oestrodial aromatised from testosterone responsible for these changes in men [73]. Taken together, these represent factors that should be controlled for in studies utilising IgG *N*-glycans.

Aside from hormones, many other blood factors are associated with IgG glycosylation. Extracellular Glc is associated with in vitro changes to IgG galactosylation and sialylation [75], with the increased availability of Gal sugar nucleotide donors (UDP-Gal) proposed as a mechanism [76]. The association of FBG to IgG glycosylation is also seen in vivo in multiple populations [11,48]. Other clinical traits found to be associated with IgG glycosylation after correcting for age include lipid profile parameters [11,48], blood pressure [11,33,48], insulin [11], the liver markers alanine aminotransferase and asparate aminotransferase [48], uric acid and urea [11,48], and fibrinogen, calcium and glycosylated haemoglobin [11].

More recently, increases in various body fat parameters were found to correlate with the increased pro-inflammatory potential of IgG. Increases in BMI, often used as a proxy to body fat, are associated with increases in IgG agalactosylation [11,77]. Further, waist circumference [11,48], the waist to hip and waist to height ratios, and dual-energy X-ray absorptiometry body fat parameters are associated with altered IgG glycosylation, with the latter explaining the most variation in the IgG glycome [78]. The importance of these findings will be validated in longitudinal studies if it is found that reducing body fat via exercise, diet or medication leads to positive changes in the IgG glycome.

Medications are associated with overall plasma glycosylation [67] and IgG-specific glycosylation [21,67]. Moreover, the effect of statin use, prevalent in many populations, was associated with IgG glycosylation [21]. Although both studies implicate medications as affecting the relative abundance of certain IgG *N*-glycan moieties, they also present inconclusive results, suggesting that this effect is so small as to not have a significant effect on the IgG glycome.

Overall, these associations may directly influence the activity of the producing B cell or alter expression of a number of “glycogenes” that encode glycosyltransferases and glycosylhydrolases [34]. In addition to the biomarkers associated with IgG glycosylation, we would expect other biomarkers within the plasma that are yet to be explored in terms of their effect on the plasma or IgG glycome. Thus, although there has been a considerable increase in our knowledge of endogenous and exogenous factors associated with altered IgG glycosylation, further investigation is still warranted.

## 9. Conclusions

It is established that genetic and other factors influence IgG glycosylation, which in turn can affect whether the IgG glycoproteins will elicit an anti-inflammatory or pro-inflammatory response. It is important to underscore these processes when considering the use of IgG *N*-glycan moieties as an indication of disease presence, progress or response to therapeutics. The integral next steps will be the undertaking of large scale and longitudinal studies that aim to further the understanding of how allelic changes, the expression of genes and the presence of other biomolecules within the cellular environment impact the IgG glycome. Indeed, altered expression of genes within any of the pathways involved in the biosynthesis of *N*-glycans, IgG FcRs or complement factors may significantly alter IgG response. Moreover, changes along the FcR or complement pathways can alter the transcription of cytokines or other biomolecules that in turn act as mediators within the immune system, such that they can regulate whether an anti-inflammatory or pro-inflammatory response is required. The translation of this knowledge will be the ability to decipher whether aberrant IgG *N*-glycan moieties within a biomarker study, particularly those of age-related chronic diseases, are indeed related to the pathophysiology of the disease or are within the limits of the natural heterogeneity of IgG glycosylation.

## Figures and Tables

**Table 1:** Summary of fragment crystallisable receptors (FcRs) ligated by IgG. Regulation: either up- or down-regulation of the expression of the FcR on a given cell. Produces: the cytokines released following binding with the given FcR. Effects: summary points from the literature.

Receptor	Classification	IgG Affinity	Cell Types	Regulation	Produces	Effects	Ref (1st Author)
FcγRI (CD64)	Type I Activating	High for IgG1, 3, 4 Low or No affinity for IgG2	Mast cells, Monocytes, Macrophages, Neutrophils, Dendritic Cells	↑ IL-10, INF-γ; ↓ IL-4	IL-6 TNF-α	↑ B Cell Differentiation, Immunoglobulin Production, Acute-Phase Protein Synthesis; ↑ ADCC, Degranulation, Phagocytosis (depends on expressing cell)—through SRC-family kinase phosphorylation; ↓ Lipo-Protein Lipase, FcγRIIIb on Neutrophils	Akira (1990) [53], Daëron (1997) [54], Lu (2015) [23], Pincetic (2014) [15], Ravetch (2001) [51], Quast (2017) [16], Rogers (2006) [55], Woolhiser (2001) [56]
FcγRIIa1/2 (CD32a)	Type I Activating	Low	Mast Cells, Monocytes, Macrophages, Neutrophils, Dendritic Cells, Eosinophils, Basophils, Platelets	↓ IL-4	TNF-α	↑ ADCC, Degranulation, Phagocytosis (depends on expressing cell)—through SRC-family kinase phosphorylation	Daëron (1997) [54], Karsten (2012) [37], Pincetic (2014) [15], Ravetch (2001) [51]
FcγRIIb1/2/3 (CD32b)	Type I Inhibitory	Low ↓ with Fuc ↑ with Gal	T Cells, NK Cells, Immature B Cells (only FcR)	↑ IL-4		↓ BCR-Induced Ca^2+^ Mobilisation, Cell Proliferation, ITAM-Regulated FcRs, Akt; Limits Autoantibody Production (on B Cells)	Karsten (2012) [50], Quast (2017) [16], Subedi (2016) [27], Unkeless (1997) [57], Vivier (1997) [58]
FcγRIIc (CD32c)	Type I Activating	Low ↓ with Fuc	Monocytes, Neutrophils, NK Cells	↓ IL-4	TNF-α	↑ ADCC, Degranulation, Phagocytosis (depends on expressing cell)—through SRC-family kinase phosphorylation	Daëron (1997) [54], Karsten (2012) [37], Pincetic (2014) [15], Ravetch (2001) [51], Subedi (2016) [27]
FcγRIIIa (CD16a)	Type I Activating	Medium ↓ with Fuc, Neu5Ac	NK Cells, Macrophages, Monocytes (10%)	↓ IL-4	TNF-α	↑ ADCC	Daëron (1997) [54], Pincetic (2014) [15], Ravetch (2001) [51], Subedi (2016) [27]
FcγRIIIb (CD16b)	Type I Activating	Low ↓ with Fuc	T Cells, Neutrophils, Macrophages, Monocytes	↑ INF-γ ↓ TNF-α		↑ Ca^2+^ Mobilisation	Fernandes (2005) [59], Hořejší (1999) [60], Pincetic (2014) [15], Ravetch (2001) [51], Subedi (2016) [27]
FcεRII (CD23)	Type II Inhibitory	? ↑ with Neu5Ac	B Cells, T Cells, Monocytes, Follicular Dendritic Cells, Macrophages			↑ Production FcγRIIb—Inhibits further activating FcγR binding	Pincetic (2014) [15], Sondermann (2013) [52]
DC-SIGN (CD209)	Type II Inhibitory	? ↑ with Neu5Ac	B Cells, Monocytes, Dendritic Cells, Macrophages	↑ IL-4, IL-33	IL-4	↑ Production FcγRIIb—Inhibits further activating FcγR binding	Pincetic (2014) [15], Schwab (2013) [4], Sondermann (2013) [52], Quast (2015) [39]

?, unknown; ↑, increased affinity/regulation/effect; ↓, decreased affinity/regulation/effect; Fuc: Core fucose; Gal: terminating galactose; Neu5Ac: terminating sialic acid; ADCC: antibody-dependent cell cytotoxicity; BCR: B-cell receptor.

**Table 2:** Summary of FcR effector responses elicited by IgG binding. IgG *N*-glycosylation of the Fc domain is directly linked to changes in affinity for FcRs that either produce more pro-inflammatory responses (ADCC) or anti-inflammatory responses (immune modulation). Anti-inflammatory activity is said to be dominant.

Effector Response	Immune Cells	Inflammation	Relation to IgG	Ref (1st Author)
Cytokines - Molecules with hormone-like function	All	Both	Altered IgG glycosylation may be linked to changes in cytokine expression	Lin (1995) [64]
Degranulation - Release of antimicrobial cytotoxic agents	Mast Cells, Basophils, Neutrophils, Eosinophils, Cytotoxic T Cells, NK Cells	Pro	↑ Fcγ-RI binding = ↑ degranulation, thus ↑ localised inflammation	Woolhiser (2001) [56]
Phagocytosis - Recognising & engulfing large particles or cells opsonised by C3b or IC, or amassed IC	Mast Cells, Basophils, Neutrophils, Eosinophils, Macrophages	Pro	↑ Fc binding can lead to ↑ localised inflammation	Quast (2014) [6], Russell (2009) [65]
ADCC - Cell lysis mediated by cytotoxic granules containing perforin & granzymes	NK Cells, Macrophages, Monocytes, Neutrophils, Eosinophils	Pro	↓ core fucosylated/sialylated IgG = ↑ Fcγ-RIIIa binding = ↑ ADCC Overall, this leads to ↑ localised inflammation/cell apoptosis	Nimmerjahn (2005) [38], Quast (2014) [6]
Immune Modulation - Upregulation of Fcγ-RIIb, which dominantly inhibits activating FcR	All	Anti	↑ sialylated IgG = ↑ Fcγ-RIIb binding Overall, this leads to ↑ anti-inflammatory activity	Pincetic (2014) [15], Schwab (2013) [4], Sondermann (2013) [52]

=, “leads to” or equal to; ↑, increased; ↓, decreased.

## Abbreviations

The following abbreviations are used in this manuscript:

IgG	Immunoglobulin G
FcR	Fragment crystallisable receptor
ADCC	Antibody-dependent cell cytotoxicity
MBL	Mannose-binding lectin
Fc	Fragment crystallisable
Fab	Fragment antibody-binding
Asn	Asparagine
Ser	Serine
Thr	Threonine
ER	Endoplasmic reticulum
Man	Mannose
GlcNAc	*N*-acetylglucosamine
Glc	Glucose
BCR	B-cell receptor
Neu5Ac	N-acetylneuraminic acid (aka sialic acid)
Fuc	Fucose
DC-SIGN	Dendritic cell-specific intercellular adhesion molecule-3-grabbing non-integrin
IC	Immune complex
ITAM	Immunoreceptor tyrosine-based activation motif
SH2	Src homology 2
PI-3K	Phosphatidylinositol 3-kinase
MAPK	Mitogen activated protein kinase
PLC-γ	Phospholipase C-gamma
PIP2	Phosphatidylinositol 4, 5-bisphosphate
IP3	Inositol 1, 4, 5-triphosphate
DAG	Diacylglycerol
ITIM	Immunoreceptor tyrosine-based inhibitory motif
SHIP	SH2 inositol 5-phosphate
PTEN	Tensin homologue
PIP3	Phosphatidylinositol-3, 4, 5-triphosphate
PKC	Protein kinase C
MAC	Membrane attack complex
BMI	Body mass index
HDL	High-density lipoprotein
LDL	Low-density lipoprotein
TC	Total cholesterol
TG	Triglycerides
FBG	Fasting blood glucose
